# A Compact Closed Genome of *Orientia tsutsugamushi* from Hainan Island, China Provides a TA763_A Reference and Reveals Repeat-Driven Remodeling

**DOI:** 10.3390/pathogens15030318

**Published:** 2026-03-16

**Authors:** Yi Niu, Yijia Guo, Zhao Xu, Siqi Chen, Liyuan Zhang, Xiuji Cui, Dachuan Lin, Kwok-Yung Yuen, Jasper Fuk-Woo Chan, Chuanning Tang, Feifei Yin

**Affiliations:** 1 Hainan Medical University-The University of Hong Kong Joint Laboratory of Tropical Infectious Diseases and Academician Workstation of Hainan Province, Key Laboratory of Tropical Translational Medicine of Ministry of Education, School of Basic Medical Sciences, Hainan Academy of Medical Sciences, Hainan Medical University, Haikou 571199, China; 2200101001@muhn.edu.cn (Y.N.);; 2 Department of Pathogen Biology, Hainan Medical University, Haikou 571199, China; 3 Department of Infectious Diseases, The Second Affiliated Hospital of Hainan Medical University, Haikou 570311, China; 4 State Key Laboratory of Emerging Infectious Diseases, Carol Yu Centre for Infection, Department of Microbiology, School of Clinical Medicine, Li Ka Shing Faculty of Medicine, The University of Hong Kong, Pokfulam, Hong Kong SAR, China; 5 Department of Infectious Diseases and Microbiology, The University of Hong Kong-Shenzhen Hospital, Shenzhen 518053, China

**Keywords:** *Orientia tsutsugamushi*, complete genome, long-read sequencing, repeat-mediated evolution, genome rearrangement, phylogeny

## Abstract

Scrub typhus, caused by the obligate intracellular bacterium *Orientia tsutsugamushi* (*O. tsutsugamushi*), remains a major public-health concern in the Asia–Pacific region. Genome-wide inference is complicated by extensive repetitive DNA and frequent genome rearrangement. We isolated *O. tsutsugamushi* HMU_001 from a scrub typhus patient on Hainan Island, China. Intracellular morphology was examined and replication was quantified in endothelial cells. Using long-read sequencing with short-read polishing, we generated a closed circular genome and performed standardized comparative analyses across all available complete *O. tsutsugamushi* genomes. HMU_001 assembled as a 1,895,724 bp genome and, among the 17 complete genomes analyzed in this study, represented the most compact genome. Repeats comprised 873,550 bp (46.08%) and included 72 RAGE loci (4 relatively complete) and 283 insertion sequences (54 intact). Repeat content varied widely and largely explained genome size differences. A core-gene phylogeny resolved four clades with partial geographic structure, while *tsa56* genotypes were only partly congruent with it. Genome synteny was generally limited across strains but markedly higher among the closest relatives, consistent with ongoing rearrangement. HMU_001 expands representation of complete *O. tsutsugamushi* genomes by adding a TA763_A lineage strain from a high-incidence island setting. Comparative analyses support a model in which repeat proliferation and decay drive genome evolution and structural remodeling.

## 1. Introduction

*Orientia tsutsugamushi* (*O. tsutsugamushi*) is an obligate intracellular α-proteobacterium in the family *Rickettsiaceae* and the causative agent of scrub typhus. Humans acquire infection primarily through the bite of infected larval trombiculid mites (chiggers) [1]. Scrub typhus has historically been considered endemic within the “tsutsugamushi triangle” in the Asia–Pacific region, but its recognized distribution has broadened over recent decades [2,3]. In addition to re-emergence in long-established endemic settings, sporadic cases and outbreaks have been reported outside this classic range [4]. Moreover, genetically divergent *Orientia* lineages, including *Candidatus Orientia chuto* and *Candidatus Orientia chiloensis*, have been described in the Middle East and South America, respectively. Emerging evidence indicates wider diversity within the genus and underscores ongoing questions about the evolution and global risk of *Orientia* sp. [5,6].

Clinically, scrub typhus typically presents as an acute febrile illness with nonspecific manifestations, such as headache, myalgia, cough, and gastrointestinal symptoms, rash, and lymphadenopathy [1]. Eschar at the site of the chigger bite is pathognomonic but is absent in a substantial fraction of patients [7]. Delayed diagnosis and treatment may lead to severe complications such as pneumonitis or acute respiratory distress syndrome, meningoencephalitis, acute kidney injury, disseminated intravascular coagulation, and myocarditis [8,9]. Laboratory confirmation relies primarily on serologic assays, such as indirect immunofluorescence assay, enzyme-linked immunosorbent assay (ELISA), and polymerase chain reaction (PCR) targeting *O. tsutsugamushi* genes such as 56-kDa type-specific antigen (*tsa56*), *tsa47*, *16S rRNA*, and *groEL* [10,11,12]. Although doxycycline and related agents are generally effective, the clinical burden remains substantial in many endemic settings, particularly where access to timely diagnostic and treatment is limited [13,14].

A long-standing challenge in scrub typhus research is the extraordinary antigenic and genetic diversity of *O. tsutsugamushi*. Early strain classification relied on serologic approaches using prototype strains (Gilliam, Karp, Kato), while molecular typing of the immunodominant *tsa56* gene is widely used for strain assignment in clinical and vector samples [15,16,17,18]. However, accumulating evidence indicates that *tsa56*-based classification only partially reflects genome-scale relationships, as virulence and phenotypic variation likely arise from distributed strain-specific determinants rather than a single locus [19,20,21,22]. Multilocus sequence typing (MLST), or concatenation of TSA56 with selected autotransporter loci such as ScaA, can improve phylogenetic resolution but remains influenced by homologous recombination and does not fully capture genome-wide architecture [20,23,24,25]. These limitations highlight the importance of genome-scale analyses for resolving evolutionary relationships in *O. tsutsugamushi*.

Whole-genome sequencing provides the most comprehensive framework for understanding *O. tsutsugamushi* diversity and for linking strain variation to phenotypes relevant to transmission, immune recognition, and disease. However, complete-genome sequencing of *O. tsutsugamushi* remains technically demanding because its chromosome is unusually large for an obligate intracellular bacterium (approximately 1.9–2.5 Mb in published complete genomes) and is densely populated by repetitive, mobile element–derived DNA [22,26,27]. These repeats are dominated by Rickettsiales amplified genetic elements (RAGEs) that resemble integrative and conjugative element systems, and by insertion sequences (IS elements) [27,28]. Repeat proliferation is accompanied by extensive pseudogenization and fragmentation, and comparative analyses have shown profound disruption of gene order among strains, consistent with ongoing repeat-mediated rearrangement [22]. Long-read sequencing has improved the ability to span repeats and generate closed assemblies [22,29], but the number of publicly available complete genomes remains limited, constraining robust phylogeographic inference and comprehensive comparisons across endemic regions and *tsa56* genotypes. Consequently, because complete genomes remain sparse and unevenly distributed across genotypes and regions, it remains unclear whether repeat burden and genome architecture vary systematically among lineages or geographic settings.

Hainan Island in southern China is an endemic setting for scrub typhus, with a mean annual reported incidence of 2.75 per 100,000 population during 2008–2023 and an overall increasing trend [30]. Recent clinical surveillance further detected *O. tsutsugamushi* in 14.51% (73/503) of patients with undifferentiated febrile illness in Hainan during 2018–2021 [31]. Despite its public-health importance, closed genomic references from this region are lacking. In this study, we isolated *O. tsutsugamushi* strain HMU_001 directly from a laboratory-confirmed scrub typhus case in Hainan from which a viable isolate was successfully recovered and propagated, enabling intracellular characterization and whole-genome sequencing. The case was also clinically well characterized, allowing interpretation of the genome in a defined clinical context. In addition, the strain belonged to the TA763_A *tsa56* lineage, for which no complete *O. tsutsugamushi* genome was available in the comparative dataset analyzed in this study. Using long-read sequencing complemented by short-read polishing, we generated a complete circular genome and performed standardized comparative analyses against all publicly available complete *O. tsutsugamushi* genomes. Our main objectives were to (i) provide a closed genome reference representing a contemporary clinical isolate from a tropical island setting, (ii) quantitatively describe variation in genome composition, repeat burden, gene duplication, and gene degeneration across the species, and (iii) place HMU_001 within a core-genome phylogeny while evaluating patterns of genome synteny and rearrangement.

## 2. Materials and Methods

### 2.1. Ethics Statement

This study was approved by the Ethics Committee of Hainan Medical University (Approval No. HYLL-2020-061). Written informed consent was obtained from the patient for participation and for publication of de-identified information.

### 2.2. Case Identification and Laboratory Confirmation

A laboratory-confirmed case of scrub typhus from Hainan Island, China identified in 2023 was included in this study. Whole blood and serum samples were collected at admission for diagnostic testing. Serum IgM and IgG antibodies against *O. tsutsugamushi* were screened using a colloidal gold immunochromatographic assay (GICA; Wantai, Beijing, China) targeting TSA56. In parallel, DNA extracted from whole blood was tested using a multiplex real-time PCR targeting the *tsa56*, *tsa47*, and *groEL* genes (Zybio, Chongqing, China), following the manufacturer′s instructions. For *tsa56* lineage assignment, a partial fragment was amplified by nested PCR [32] and its sequence was obtained by Sanger sequencing. Genotypes were assigned based on sequence similarity and phylogenetic placement. Clinical and laboratory information was retrieved from the patient’s medical record.

### 2.3. Isolation and Culture of O. tsutsugamushi from Blood Clot

Approximately 1 mL of blood clot was mechanically disrupted with 3 mm zirconia beads (4000 rpm for 15 s, three cycles). The suspension was inoculated onto 80–90% confluent L-929 monolayers in 25 cm^2^ flasks. Inoculated cells were maintained at 35 °C with 5% CO_2_ in Minimum Essential Medium (MEM; Gibco, Grand Island, NY, USA) supplemented with 2% horse serum (Gibco, Grand Island, NY, USA) and non-essential amino acids (NEAA; Gibco, Grand Island, NY, USA). Cultures were examined daily by light microscopy, and the culture medium was replaced at 48 h post-inoculation and subsequently as needed. Successful infection was assessed by the development of cytopathic effects (CPE) and Giemsa staining following standard procedures. When CPE involved ≥90% of the monolayer (as estimated by microscopy), infected cells were passaged at a 1:2 split ratio by scraping and transferring the cell suspension onto fresh L-929 monolayers.

### 2.4. Immuno-Electron Microscopy

L-929 cells were infected with *O. tsutsugamushi* at an input ratio equivalent to a multiplicity of infection (MOI) of 100 bacterial genome copies per cell, calculated from the genome DNA load and the host–cell count at inoculation. At 3 days post infection (dpi), cells were fixed in 2.5% glutaraldehyde, embedded in resin, sectioned into 70–80 nm slices and mounted on nickel grids. Sections were incubated with rabbit anti-TSA56 polyclonal antibody (Sangon Biotech, Shanghai, China; 1:5000), followed by incubation with 10 nm colloidal gold-conjugated goat anti-rabbit IgG secondary antibody (Sigma-Aldrich; St. Louis, MO, USA, 1:2000). Labeled sections were examined using a Hitachi HT7800 transmission electron microscope (Hitachi High-Tech, Tokyo, Japan).

### 2.5. Growth Kinetics by qPCR

Human umbilical vein endothelial cells (HUVECs) were seeded in 12-well plates at 1 × 10^5^ cells per well and incubated overnight to allow adherence. *O. tsutsugamushi* was purified from infected L-929 cells as described previously [33]. Briefly, infected L-929 cells were disrupted by 1 mm zirconia beads (3000 rpm for 15 s, three cycles) and then centrifuged at 50× *g* for 5 min to remove cell debris. The supernatant was subsequently centrifuged at 20,000× *g* for 10 min to pellet bacteria. The bacterial pellet was resuspended in new culture medium and used to infect HUVEC monolayers at an MOI of 100. Infected HUVECs were maintained at 35 °C with 5% CO_2_ in Dulbecco’s Modified Eagle Medium (DMEM; Gibco, USA) supplemented with 2% fetal bovine serum (Gibco, USA). Cells were harvested at 4 h post infection and then daily from 1 to 9 dpi. Bacterial growth curves were generated by plotting the genome copy number per well over time. For quantifying the bacteria, total genomic DNA was extracted from each well using the QIAamp DNA Mini Kit (Qiagen, Hilden, Germany) and quantified by real-time qPCR targeting *tsa56* using the primers: 5′-GGCCAAGTTAAACTCTATGCTGA-3′ (forward) and 5′-CATTAATTGCTACACCAAGTGC-3′ (reverse), yielding an approximately 163 bp amplicon. Reactions were performed with TB Green^®^ Premix Ex Taq™ II (Takara, Kyoto, Japan) on an BIO-RAD CFX96 Real-Time PCR System (Bio-Rad, Hercules, CA, USA) under the following cycling conditions: 95 °C for 30 s; 40 cycles of 95 °C for 5 s and 60 °C for 30 s; followed by melt-curve analysis. Bacteria genome copy numbers were calculated from a plasmid DNA standard curve (10^1^–10^8^ copies/reaction). The experiment was performed independently three times. Bacterial doubling time (d.t.) was calculated as d.t. = t × 0.301/log_10_ (N_2_/N_1_), where t represented the elapsed time and N_2_/N_1_ was the fold change in bacterial genome copies between two time points. Bacteria load at each time point and d.t_min_ were represented by mean ± SD.

### 2.6. Genomic DNA Preparation and Whole-Genome Sequencing

A total of 1 × 10^7^ L-929 cells were infected with *O. tsutsugamushi* at an MOI of 100 and incubated at 35 °C with 5% CO_2_. At 5 dpi, cells were harvested and mechanically disrupted with 1 mm zirconia beads (3000 rpm for 15 s, three cycles). The lysate was centrifuged at 50× *g* for 10 min to remove host–cell debris, and bacteria in the supernatant were further pelleted at 20,000× *g* for 30 min. The pellet was washed three times with phosphate-buffered saline (PBS) and genomic DNA was extracted using the QIAamp DNA Mini Kit. High molecular weight DNA (≥50 kb) was used for long-read sequencing. A SMRTbell library (Pacific Biosciences, Menlo Park, CA, USA) was prepared and genomic DNA was sheared to an average size of approximately 15 kb, followed by end-repair and SMRTbell adapter ligation. Fragments ≥ 15 kb were size-selected on a BluePippin instrument (Sage Science, Beverly, MA, USA) and sequenced on a PacBio Revio Single-Molecule Real-Time (SMRT) Sequencing system (Pacific Biosciences, Menlo Park, CA, USA). Circular consensus sequencing (CCS) was performed using PacBio ccs software to generate high-fidelity (HiFi) reads (long reads). In addition, a short-read paired-end library (input DNA ≥ 0.2 µg) was prepared using the Watchmaker DNA Library Prep Kit (7K0019-096; MGI Tech, Shenzhen, China) and sequenced on an MGI DNBSEQ-T7 Sequencing System (MGI Tech, Shenzhen, China) to generate 150-bp paired-end reads (short reads), following the manufacturers’ instructions.

### 2.7. Genome Assembly, Annotation and Comparative Analysis

PacBio HiFi reads were filtered for host contamination by mapping to the mouse reference genome (GCF_000001635.27) using minimap2 v2.29, and unmapped reads were de novo assembled with hifiasm v0.12 (default parameters) [34]. The circular chromosome was finalized with Circlator v1.5.5 [35], including rotation to start at *dnaA*. Paired-end short reads were trimmed with Trimmomatic v0.33 and screened against the mouse genome with Bowtie2 v2.2.4. Non-host short reads were mapped to the HiFi assembly with BWA-MEM2 v2.2.1, and three iterative rounds of polishing were performed using Pilon v1.22 to correct residual base-level errors and improve consensus accuracy of the assembly [36].

Assembly quality was further evaluated by examining sequencing depth, consensus accuracy, and genome circularization. After host-read removal, effective sequencing depth at each genome position was calculated using samtools depth. Sequencing depth distribution histogram and genome-wide sequencing depth profile plot were examined to assess coverage distribution and mapping consistency. Regions showing reduced depth were inspected to evaluate potential mapping bias associated with repetitive genomic regions. Base-level alignment statistics, including mismatch and indel rates, were calculated from samtools stats reports. To validate circularization, a junction-containing reference was generated by appending the terminal 20 kb of the assembly to the genome start, and both long-read and short-read datasets were remapped to quantify reads spanning the circularization junction.

Protein-coding sequences (CDSs) were predicted and annotated with Prokka v1.14.6 [37] using a custom database derived from NCBI PGAP annotations for previously sequenced *O. tsutsugamushi* genomes. rRNA and tRNA predictions were refined using Infernal v1.1.5 [38] and tRNAscan-SE v2.0.9 [39], respectively. Pseudogenes were predicted with Pseudofinder v1.1.0 [40] under the following criteria: (i) CDSs were searched against the NCBI nr database, and CDSs shorter than 90% of the mean length of their top 150 BLAST homologs were classified as truncated genes. This threshold was selected empirically during method optimization by comparing the effects of several alternative parameter settings and was used as a practical screening cutoff in our dataset. Our pseudogene analysis was intended as a conservative comparative screen for likely truncation- and fragmentation-associated pseudogenization rather than optimization of a locus-specific functional cutoff. The use of mean homolog length followed the native implementation of the workflow employed here. Comparison with the mean length of the top 150 BLAST homologs was used to provide a stable reference length distribution while reducing the influence of annotation artifacts or atypical individual matches. (ii) Adjacent CDSs sharing 30% of their hits, or CDSs sharing hits with intergenic regions, were classified as split genes. Because degraded ancestral genes in *O. tsutsugamushi* are frequently represented as short CDSs or multiple adjacent fragments, original disruptive mutations such as internal stop codons or frameshifts would not be reliably recovered; therefore, we relied primarily on homology-based evidence of truncation and fragmentation. This approach was intended to distinguish likely truncation events from natural homolog length variation by combining a conservative length threshold with comparison against a broad homolog set. CDSs longer than their homolog length distribution and pseudogene candidates located entirely within intergenic regions were not included. COG functional categories were assigned with eggNOG-mapper v2.1.12 [41]. Sixteen publicly available complete *O. tsutsugamushi* genomes (Table 1) were re-annotated using the same pipeline; genomes not starting at dnaA were rotated using Circlator (fixstart).

Orthogroups were inferred with OrthoFinder v3.0.1b1 [42], and single-copy core genes were defined as genes in orthogroups presented in all 17 genomes with exactly one ortholog per genome. Concatenated amino acid sequences of single-copy orthologs aligned with MAFFT v7.525 were used to infer a maximum-likelihood phylogeny in RAxML v8.2.12 [43] (PROTGAMMALG, 1000 bootstraps; visualized in FigTree v1.4.4). For comparison, partial *tsa56* nucleotide sequences (approximately 483 bp) were aligned with ClustalW v2.1 and analyzed by RAxML (GTRGAMMA, 1000 bootstraps), rooted on the Boryong strain. MLST sequence types (STs) of the 17 strains analyzed in this study were inferred from nucleotide sequences of seven housekeeping genes (*gpsA*, *mdh*, *nrdB*, *nuoF*, *ppdK*, *sucB*, and *sucD*) extracted from genome data according to the allele profiles defined in the PubMLST *O. tsutsugamushi* database (https://pubmlst.org/organisms/orientia-tsutsugamushi/ (accessed on 10 March 2026)) [44].

Repeat regions were identified by BLASTn v2.16.0+ self-alignment of the chromosome (identity ≥ 90%, alignment length ≥ 50 bp, E < 1 × 10^−5^). Self-hit alignments were removed, and overlapping coordinates were merged using BEDTools v2.31.1 to define nonredundant repeat intervals. Repeat content was calculated as the total base number of merged intervals. Repeat genes were defined as genes with at least one genomic homolog detected by BLASTn (identity ≥ 90%, query coverage ≥ 80%; E-value < 1 × 10^−5^). The proportions of repeat genes in each COG categories, and the proportions of pseudogenes in repeat genes and non-repeat genes, were calculated and compared among the 17 genomes. In addition, pseudogenization rates in repetitive and non-repetitive genes were compared across the 17 genomes using a paired Wilcoxon signed-rank test; *p* < 0.05 was considered statistically significant.

RAGEs in the HMU_001 genome were identified based on published structural criteria and manual curation, focusing on genomic regions containing ICE-associated modules (integrase genes and conjugation-associated F-T4SS gene clusters), transposase/reverse transcriptase genes together with associated cargo genes [27,28]. Following published criteria [28], we distinguished “complete RAGE”, defined as structurally complete elements retaining the full set of intact mobilization gene categories in overall canonical organization, from “complete RAGE with truncated or split genes”, in which the full set of mobilization gene categories is present but at least one mobilization gene is truncated or split. Loci lacking key mobilization components were classified as incomplete. To further assess gene integrity within RAGEs, conserved domains were evaluated using SMART v10.0 as described previously [28], and RAGE-associated genes were categorized as intact if they contained all key functional domains. ISs in HMU_001 were detected by BLASTn searches against the ISfinder database v2.16.0 (identity ≥ 90%, E-value <1 × 10^−5^), with manual verification of boundaries where needed. A complete IS was defined as an element containing a full-length transposase gene.

Pairwise synteny across the 17 genomes was assessed using MUMmer v4.0.1 (nucmer, MUM mode with a minimum cluster length of 500 bp to reduce noise from repetitive regions) [45]. Syntenic blocks ≥ 2000 bp (identity ≥ 90%) were visualized in R v4.4.3 (ggplot2) or Easyfig v2.2.5. For each pairwise comparison, the syntenic ratio was calculated as the total length of syntenic blocks divided by the query genome length, and the number of syntenic blocks was recorded. When calculating length of syntenic blocks, adjacent syntenic blocks that were slightly overlapped were merged using BEDTools to prevent repeated calculation of bases at the junctions, which had almost no impact on estimation of syntenic ratios.

### 2.8. Data Availability

The complete genome sequence of *O. tsutsugamushi* strain HMU_001 is available in NCBI (accession: CP191261, BioProject: PRJNA1258383, BioSample: SAMN48320726). Raw reads were deposited in SRA (SRR33649153 (PacBio), SRR33649152 (MGI)).

## 3. Results

### 3.1. Clinical Presentation and Laboratory Findings

A 57-year-old female forestry worker living in southern Hainan Island was admitted to the Second Affiliated Hospital of Hainan Medical University in July 2023. The patient reported a five-day history of fever after working in tropical rainforest areas. On admission, she had fever of up to 39.5 °C and hypoxemia with SpO_2_ of 89% at room air. Physical examination revealed a 1.0 cm × 0.5 cm eschar at the left inguinal region. Chest computerized tomography scans of the thorax showed scattered bilateral pulmonary infiltrates. Laboratory tests showed leukocytosis with marked neutrophilia, elevated inflammatory markers, coagulation abnormalities, mild hepatic and renal dysfunction, and hypoalbuminemia (Table S1). Arterial blood gas testing indicated metabolic acidosis with elevated lactate (Table S1).

Serum IgM and IgG against *O. tsutsugamushi* were positive. Quantitative PCR was positive with an estimated bacterial load of 2.68 × 10^5^ copies/mL. Genotyping of a partial *tsa56* fragment assigned the strain to the TA763_A lineage. The patient was diagnosed with scrub typhus complicated by pneumonia, hypoxemia, acid-base imbalance, and hepatic and renal dysfunction. Doxycycline (100 mg intravenously every 12 hourly) was administered and that patient improved with defervescence, improved oxygenation, and normalization of laboratory tests in the ensuing 48 h (Table S1). The patient was discharged after 5 days of hospitalization.

### 3.2. Bacterial Morphology and Growth Kinetics

**Figure 1:**
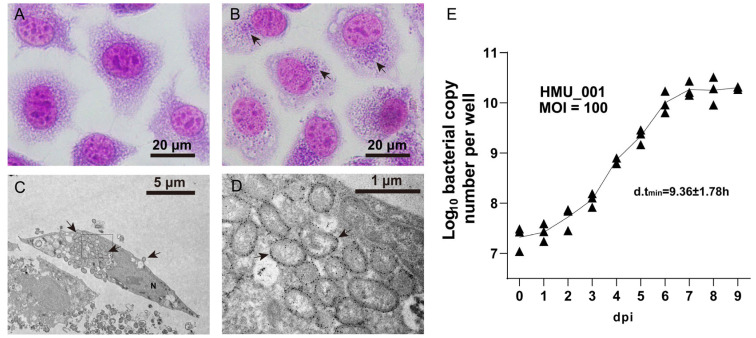
Morphology and intracellular growth of *Orientia tsutsugumashi* (*O. tsutsugamushi*) HMU_001. (**A**) Giemsa-stained uninfected L-929 cells. (**B**) Giemsa-stained L-929 cells infected with HMU_001 showing perinuclear clusters of bacteria (arrows). Scale bars, 20 μm. (**C**) Immunogold transmission electron micrograph of an infected cell showing intracellular bacteria concentrated near the nucleus (N); gold particles indicate TSA56 labeling. The boxed region is enlarged in (**D**). Scale bar, 5 μm. (**D**) Higher-magnification view showing spherical to short rod-shaped organisms with a double-membrane envelope and surface-associated TSA56 immunogold particles. Scale bar, 1 μm. (**E**) Replication kinetics of HMU_001 in human umbilical vein endothelial cells measured by qPCR. Points show mean ± SD genome copies per well from three independent biological replicates at the indicated days post infection. The minimum doubling time was estimated from the exponential growth interval between 3 and 4 dpi.

*O. tsutsugamushi* was isolated from the patient’s blood clot in L-929 cell monolayers and designated as strain HMU_001. Bacterial morphology and intracellular localization were examined by Giemsa staining and immuno-electron microscopy. On Giemsa staining, infected cells showed dense perinuclear clusters of purple granular organisms, whereas uninfected controls displayed pale-purple reticular cytoplasm (Figure 1A,B). Immuno-electron microscopy revealed intracellular bacteria that were spherical to ovoid or short rods, approximately 0.3–0.5 × 0.8–1.5 µm, and predominantly clustered near the nucleus. Each organism displayed a double-membrane envelope with a prominent electron-lucent intermembrane space and a loose, heterogeneous electron-dense internal matrix. Sparse immunogold labeling targeting TSA56 was observed on the bacterial surface by electron microscopy, with an uneven distribution along the envelope (Figure 1C,D). Occasionally, membrane-enclosed organisms were observed near the cell periphery, showing a budding-like appearance; however, this finding is descriptive only and does not imply a specific replication mechanism (Figure 1C).

To quantify replication, we infected HUVECs in 12-well plates with purified bacteria at an MOI of 100 and measured bacterial genome DNA by qPCR over time. At 4 h post-infection (defined as day 0), the bacterial load was (2.29 ± 1.05) × 10^7^ genome copies per well. The bacterial burden increased modestly during 0–2 dpi, then entered a clear exponential phase between 3–6 dpi, reaching (1.09 ± 0.56) × 10^10^ copies per well. Growth slowed thereafter and plateaued between 7–9 dpi at (1.92 ± 0.69) × 10^10^ genome copies per well, representing an approximately 1000-fold increase over the initial level. The steepest increase occurred between 3 and 4 dpi, corresponding to a minimal doubling time of 9.36 ± 1.78 h (Figure 1E).

### 3.3. Genome Composition and Functional Overview of O. tsutsugamushi HMU_001

We conducted genome sequencing of HMU_001 using both long-read and short-read platforms. Long-read sequencing generated 1,356,103 high-fidelity reads (26.30 Gb; mean length 19,391 bp) of which 1,076,959 reads (79.42%; 21.52 Gb) remained after host reads removal. Short-read sequencing produced 164,238,400 paired-end reads (24.64 Gb); after quality filtering and host reads removal, 105,101,742 reads (63.99%; 15.71 Gb) were retained. Filtered reads mapped to the final assembly at a mean depth of 11,337× (long reads) and 8146× (short reads), with minimum depth of 7515× and 503×, respectively. Re-examination of the depth distribution showed that the relatively low short-read depth was confined to within ~100 bp of the start/end positions of the linearized circular assembly, whereas coverage across the remainder of the genome was highly uniform; 95% of genomic sites showed short-read depths between 6784× and 9522×. This discrepancy is therefore best explained by a boundary mapping effect rather than widespread uneven coverage associated with the repetitive genome structure. The mismatch rate was 0.1738% for long reads and 0.0417% for short reads, while the indel rate was 0.1403% for long reads and 0.0032% for short reads. These low alignment error rates support the high base-level accuracy of the final assembly. In addition, 10,507 long reads spanning the circularization junction provided strong support for successful genome circularization. To assess within-isolate heterogeneity and exclude multiple genotypes, we mapped the host-filtered short reads back to the finalized assembly and examined allele frequencies across genome-wide SNP sites. The allele-frequency distribution was unimodal with a dominant peak near fixation and no secondary peak, supporting a single predominant genotype for HMU_001. Consistently, Sanger sequencing chromatograms of *tsa56* from serial passages showed clean single peaks at informative positions, indicating genotype stability during in vitro propagation.

**Figure 2:**
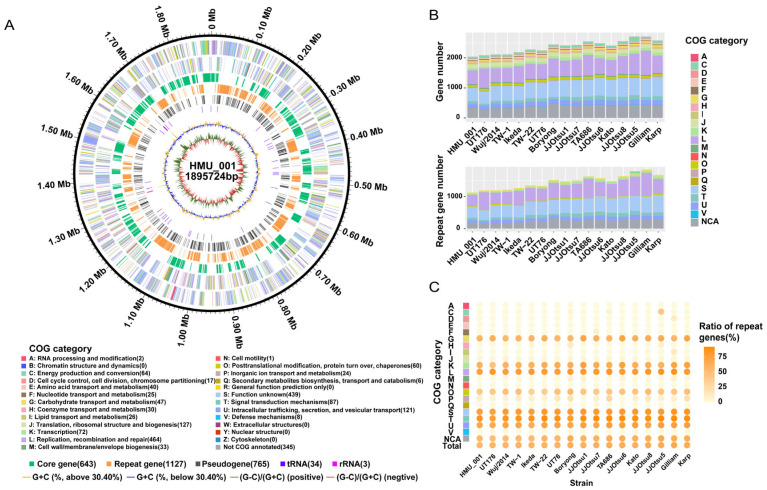
Genome features and functional profiles of HMU_001 in the context of available complete *O. tsutsugamushi* genomes. (**A**) Circular map of the HMU_001 genome. Rings from outside to inside show genome coordinates; COG functional categories for coding sequences on the forward and reverse strands; core genes defined as single-copy orthogroups present in all 17 genomes; repeat genes defined as genes with at least one homolog elsewhere in the same genome; pseudogene candidates; rRNA and tRNA genes; GC content; and GC skew. (**B**) Counts of genes assigned to each COG category across 17 complete genomes, shown for all genes (**top**) and for repeat genes only (**bottom**). (**C**) Proportion of repeat genes within each COG category across genomes. NCA, Not COG annotated.

The HMU_001 genome comprises a single circular chromosome of 1,895,724 bp with a GC content of 30.40%. Genome annotation predicted 2075 genes, including 2038 CDSs, 3 rRNA genes, and 34 tRNA genes (Figure 2A, Table S2). Among the 17 complete *O. tsutsugamushi* genomes analyzed in this study, HMU_001 had the smallest genome size and the fewest predicted genes. Using Pseudofinder, we identified 765 pseudogene candidates derived from 944 CDSs, corresponding to 46.32% of the 2038 predicted CDSs (Figure 2A). These included 587 truncated CDSs (<90% of the average length of homologs) and 178 merged pseudogenes consolidated from 357 adjacent CDSs or intergenic regions sharing >30% BLAST hits (Figure 2A, Tables S2 and S3). Summary of currently available *O. tsutsugamushi* strains and their genomes were shown in Table 1.

Functional classification using eggNOG assigned COG categories to 1693 CDSs (83.07%). The most represented category was “L: Replication, recombination, and repair” (464 genes), accounting for 22.77% of the total number of CDSs, followed by “S: Function unknown” (439 genes; 21.54%). Other common categories included “J: Translation, ribosomal structure and biogenesis” (127 genes; 6.23%) and “U: Intracellular trafficking, secretion, and vesicular transport” (121 genes; 5.94%). There were 345 CDSs (16.9%) that could not be placed into any COG category (Figure 2A, Table S2). Across the 17 complete genomes, all strains shared the same overall set of functional categories (Figure 2B; Table S4). Despite its smaller genome, HMU_001 showed an overall functional profile comparable to other complete genomes. Strain differences seemed driven mainly by variation in gene counts within specific categories.

### 3.4. Repeated Sequence Analysis

**Figure 3:**
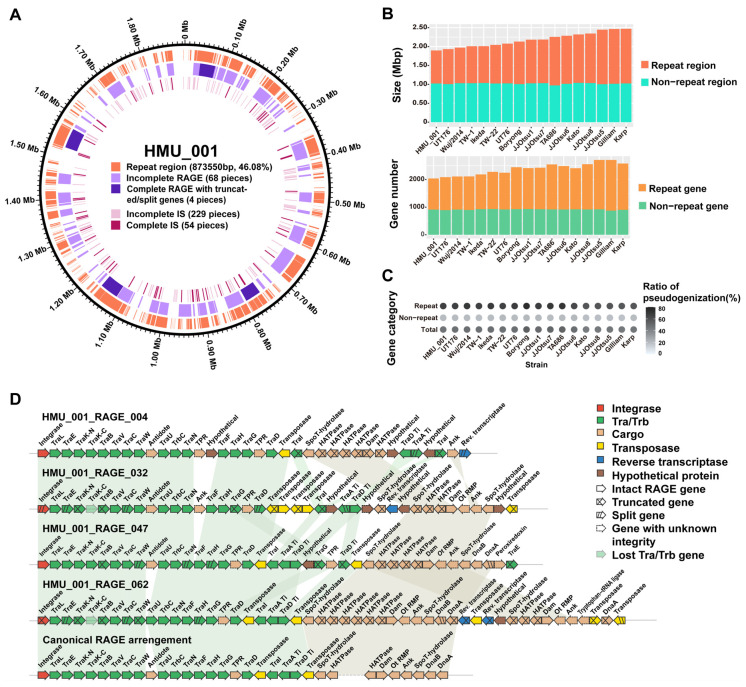
Repeat content and RAGE organization in HMU_001 and across complete *O. tsutsugamushi* genomes. (**A**) Distribution of repeat intervals and mobile element loci in the HMU_001 genome, including Rickettsiales amplified genetic elements (RAGEs) and insertion sequences (ISs); intact and fragmented elements are indicated as shown. (**B**) Sizes of repeat and non-repeat regions (top) and corresponding gene counts (bottom) across 17 complete genomes. (**C**) Pseudogenization ratios for repeat genes, non-repeat genes, and all genes across genomes. (**D**) Gene organization of the four most complete RAGE loci in HMU_001 compared with a canonical RAGE arrangement. Genes are color-coded by module (integrase, tra/trb genes, cargo genes, transposases, reverse transcriptases, and hypothetical proteins). Disrupted genes are indicated by split or degenerated arrows, and shaded links indicate homologous segments between loci.

Repetitive sequences in HMU_001 totaled 873,550 bp, accounting for 46.08% of the genome. Loci of BEDTools merged repetitive sequences were shown in Figure 3A and Table S5. Across all 17 complete *O. tsutsugamushi* genomes, the nonrepetitive (“unique”) fraction was highly consistent at ~1.00 Mb and varied by no more than 0.04 Mb, with TA686 showing a slightly smaller unique length of 0.97 Mb. In contrast, repeat content varied widely, ranging from 0.87 Mb in HMU_001 to 1.45 Mb in Gilliam (Figure 3B, Table S6). Variation in size of repetitive sequences largely accounted for differences in total genome size, supporting repeat-driven genome expansion within the species.

We then classified genes as repetitive if they had at least one additional homolog elsewhere in the same genome. HMU_001 contained 1127 repetitive genes, with the remaining 911 genes classified as non-repetitive (Table S2). Across the 17 genomes, non-repetitive gene numbers were relatively stable (typically ~900; 872 in Gilliam to 932 in TW-22), whereas repetitive gene numbers varied markedly (1127 in HMU_001 to 1837 in Gilliam), indicating that inter-strain gene-content differences are driven primarily by variable expansion of multi-copy gene families (Figure 3B, Table S7). Functionally, repetitive genes were strongly enriched in a subset of COG categories: most notably “T: Signal transduction mechanisms” (repeat-gene ratios 76.27–92.42% across strains), with “S: Function unknown” and “L: Replication, recombination and repair” also predominantly repetitive (>70% in most genomes). In contrast, most housekeeping and metabolic categories showed low duplication, with “C: Energy production and conversion” being essentially non-repetitive in nearly all genomes except JJOtsu5, where category-specific expansion (largely annotated as hypothetical proteins) inflated the repetitive fraction (Table S4). Thus, differences between strains seemed largely quantitative (repetitive genes expansion within categories) rather than qualitative (gain/loss of functional categories).

We further compared pseudogenization rates between repetitive and non-repetitive genes. Across the 17 genomes, the proportion of pseudogene candidates was consistently lower among non-repetitive genes (median, 19.98%; IQR, 1.09%) than among repetitive genes (median, 67.02%; IQR, 11.33%) (Figure 3C, Table S7). This difference was significant across genomes (paired Wilcoxon signed-rank test, *p* < 0.001), consistent with more frequent truncation or degeneration among repetitive genes, which also showed greater variability in pseudogenization rates.

The repeat landscape of HMU_001 was dominated by mobile genetic elements, particularly RAGEs and insertion sequences (ISs), providing a mechanistic basis for repeat accumulation and genome plasticity. We identified 72 RAGEs in HMU_001 (Figure 3A; Table S8). Using the criteria defined in the Materials and Methods, no complete RAGE was identified in HMU_001; instead, four loci were classified as complete RAGE with truncated or split genes, indicating that although structurally complete RAGE-like regions were present, none retained fully intact mobilization genes (Figure 3D). Overall, HMU_001 RAGEs showed substantial degeneration, including truncation, fragmentation, and loss of canonical mobilization modules, and many were reduced to only a few residual genes. Nevertheless, 22 RAGE loci retained recognizable canonical structural features (Figure S2). Importantly, at least one intact copy of each mobilization gene category was present in the HMU_001 genome, although these genes were dispersed across loci rather than organized in a contiguous module. IS elements were also abundant and dispersed across the chromosome. We detected 283 IS elements, including 54 apparently complete ones with intact transposase genes, while the remainder were truncated or degenerated (Figure 3A, Table S9).

### 3.5. Orthologous Groups, and Phylogenetic Analysis and MLST Classification

**Figure 4:**
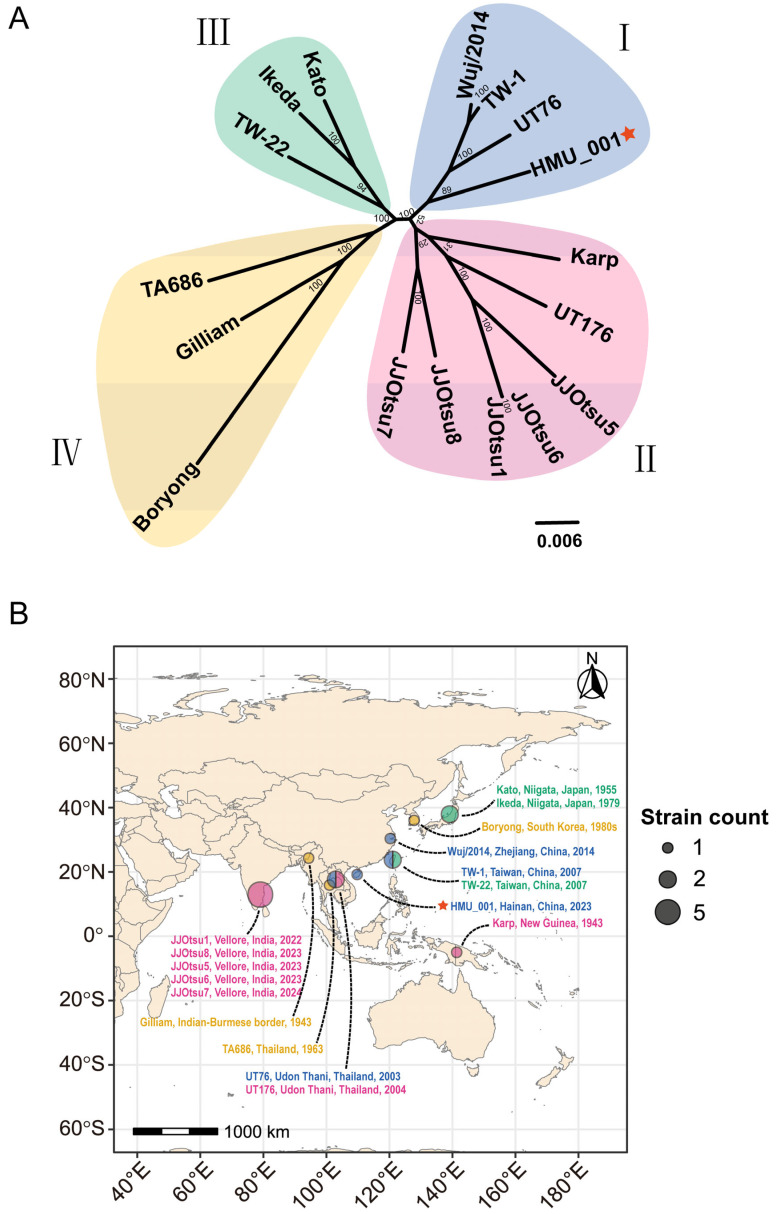
Core-genome phylogeny and sampling geography of 17 complete *O. tsutsugamushi* genomes. (**A**) Maximum-likelihood phylogeny inferred from a concatenated alignment of 643 single-copy core genes. Clades I–IV are shaded, and HMU_001 is indicated by a red star. (**B**) Map showing sampling locations of the 17 strains, colored by core-genome clade; symbol size indicates the number of genomes from each location.

Across the 17 genomes, a total of 40,325 genes were analyzed. Of these, 38,512 genes (38,512/40,325, 95.50%) clustered into 3927 orthogroups, while 1813 genes (1813/40,325, 4.50%) remained unassigned. Among the orthogroups, 343 were species-specific, comprising 1351 genes (3.35% of all genes). A core set of 664 orthogroups was present in all genomes, including 643 single-copy orthogroups (Table S2). A maximum-likelihood tree based on a concatenated alignment of the 643 single-copy core genes separated the 17 genomes into four clades (Figure 4A). HMU_001 grouped with Wuj/2014, TW-1, and UT76 in Clade I. Clade II comprised JJOtsu1, JJOtsu5, JJOtsu6, JJOtsu7, and JJOtsu8 (isolated in Vellore, India) clustered with UT176 and Karp. TW-22, Kato, and Ikeda formed Clade III, whereas TA686, Gilliam, and Boryong formed distinct branches within Clade IV. Notably, JJOtsu6 and JJOtsu1 were nearly identical, and Wuj/2014 and TW-1 also showed very close genetic relatedness.

Mapping clades to sampling locations suggested geographic structure with partial overlap (Figure 4B). Clade I comprised genomes from southeastern coastal and offshore islands of China and Thailand. Clade II encompassed genomes from South Asia (India and Thailand) and Southeast Asia (New Guinea). Clade III included genomes from regions in Northwest Pacific. Clades I to III exhibited relatively localized distributions. In contrast, Clade IV showed a broader distribution spanning Korea, Thailand, and the India–Myanmar border region, consistent with its greater phylogenetic divergence. The *tsa56* genotypes, the most widely used scheme for *O. tsutsugamushi* strain classification, did not fully align with the core-genome clade structure. In Clade I, all strains were identified as Karp_A by *tsa56* genotype except HMU_001 (TA763_A). Clade II contained a mixture of Karp_A, Karp_B, Karp_C, Kato_A, and TA763_B genotypes. Clade III included Kato_A, Kato_B, and JG_A/Gilliam genotypes. Clade IV comprised Shimokoshi, Gilliam, and Boryong/Karp genotypes (Table 1; Figure S3).

Sequence types inferred from the seven housekeeping loci are summarized in Table 1, and detailed allele profiles are provided in Table S10. Overall, the 17 strains showed substantial MLST diversity. JJOtsu6 and JJOtsu1 shared identical allele profiles at all seven loci and were both assigned to ST132. Wuj/2014 and TW-1 shared identical alleles at six of seven loci and differed only at nuoF. These highly similar MLST profiles were consistent with their close relationships in the core-genome phylogeny. Several strains, including HMU_001, JJOtsu8, Wuj/2014, TW-1, TW-22, and TA686, had seven-locus profiles that had not been assigned official STs in the PubMLST database at the time of analysis; for these strains, the nearest STs were provided in Table S10.

### 3.6. Genome Synteny Analysis

**Figure 5:**
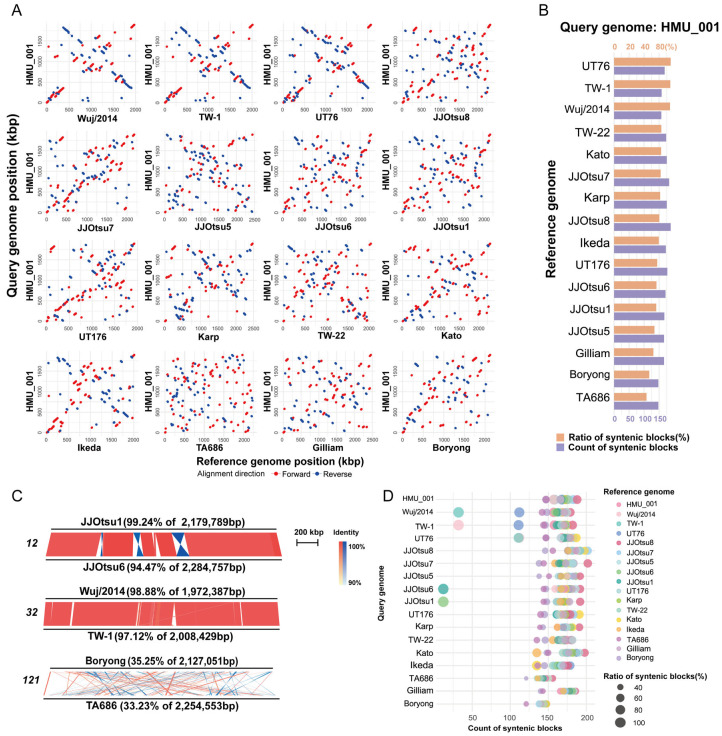
Genome synteny and structural rearrangement among complete *O. tsutsugamushi* genomes. (**A**) MUMmer dot plots comparing HMU_001 (query; y-axis) with each of the other 16 genomes (reference; x-axis). Forward and reverse alignments are shown in red and blue, respectively. Each scale mark represents 250 kbp. (**B**) Summary of synteny between HMU_001 and each reference genome, shown as the fraction of HMU_001 sequence in syntenic blocks and the number of syntenic blocks identified. (**C**) Representative pairwise comparisons illustrating high and low synteny. Numbers at left indicate the number of syntenic blocks; percentages indicate the fraction of each genome covered by syntenic blocks. (**D**) Pairwise synteny across all genome pairs. Bubble plots show the number of syntenic blocks (x-axis) against query genome (y-axis); bubble size indicates the syntenic fraction of the query genome and color indicates the reference genome.

Genome-wide synteny between HMU_001 and the other 16 complete genomes was shown in Figure 5A. Overall, HMU_001 showed limited synteny with most strains. However, relatively higher synteny was observed with Wuj/2014, TW-1, and UT76, consistent with their clustering with HMU_001 in the core-genome phylogeny. Quantitative comparisons of syntenic blocks (Figure 5B, Table S11) showed that HMU_001 shared the largest proportion of syntenic sequence with UT76 (81.97%), TW-1 (81.43%), and Wuj/2014 (81.00%). Correspondingly, HMU_001 aligned in 168, 158, and 157 syntenic blocks with UT76, TW-1, and Wuj/2014, respectively. The lowest synteny with HMU_001 was observed for TA686 and Boryong, which belong to the most distant Clade IV: only 46.93% and 50.97% of the HMU_001 genome aligned to these strains, respectively, across 147 syntenic blocks in each comparison.

Pairwise synteny analysis across all 17 genomes further confirmed that most genome pairs showed limited collinearity, whereas a small number of closely related genomes retained extensive synteny. The closest pair in the phylogeny, JJOtsu6 and JJOtsu1, showed near-identical genome organization with only minor inversions and small differences. Wuj/2014 and TW-1 likewise exhibited substantially higher synteny than more distantly related pairs such as Boryong and TA686 (Figure 5C). Syntenic block counts and aligned proportions for all genome pairs are provided in Table S11 and summarized in a bubble plot (Figure 5D). Pairs with very high syntenic proportions typically showed fewer, larger pieces of blocks (JJOtsu6–JJOtsu1: 94.47% in JJOtsu6 and 99.24% in JJOtsu1 across 12 blocks; Wuj/2014–TW-1: 98.88% in Wuj/2014 and 97.12% in TW-1 across 32 blocks). As the proportion of syntenic sequence decreased, block number generally increased and then declined. At the low-synteny extreme, Boryong and TA686 shared the lowest syntenic proportions (35.25% in Boryong and 33.23% in TA686), but without exhibiting the highest block count (121 blocks).

## 4. Discussion

In this study, we isolated *O. tsutsugamushi* HMU_001 from a scrub typhus patient in Hainan, China, and generated a closed circular genome using long-read sequencing complemented by short-read polishing, thereby adding a complete genomic reference from a tropical island setting of ongoing public-health importance. HMU_001 provided a complete genome for the TA763_A *tsa56* lineage and, among the 17 complete genomes analyzed in this study, represented the most compact genome. Notably, this compaction reflects reduced repeat burden rather than contraction of the ~1.00 Mb nonrepetitive backbone, reinforcing a central feature of *O. tsutsugamushi* evolution in which genome size variation is primarily repeat-driven while core content remains comparatively stable. Using standardized re-annotation and genome-wide comparisons, we further showed that repeats largely derived from genes associated with “Signal transduction mechanisms”, “Function unknown” and “Replication, recombination and repair”, consistent with the gene function of mobile elements, including RAGEs and IS elements. These elements were presumed to be closely related with pervasive disruption of gene order, yielding minimal synteny across most genome pairs but substantial collinearity among the closest relatives. A core-genome phylogeny based on 643 single-copy genes resolved four clades with partial geographic structure and placed HMU_001 with Wuj/2014 from Zhejiang and TW-1 from Taiwan. In contrast, *tsa56* genotypes were only partly congruent with genome-wide relationships. Together, these findings provide a framework linking repeat proliferation, genome rearrangement, and phylogenetic inference, strengthening the genomic basis for interpreting typing and evolution in this highly plastic, neglected intracellular pathogen.

The epidemiologic context on Hainan Island underscores the value of closed genomes derived from contemporary clinical isolates. Scrub typhus remains as an endemic public health concern in this tropical island setting, particularly among people with frequent outdoor exposure. A spatiotemporal analysis reported 4300 cases during 2008–2023, corresponding to a mean annual reported incidence of 2.75 per 100,000 and an overall upward trend [30]. Studies of undifferentiated fever further suggest substantial exposure and ongoing transmission. In a four-hospital survey of 680 patients, the seropositivity of IgM, IgG, and PCR was 23.97%, 36.62%, and 20.88%, respectively, and 32.79% met diagnostic criteria for scrub typhus, with a clear autumn peak [19]. Scrub typhus in Hainan also occurs within a broader rickettsial landscape: an outpatient cohort detected *O. tsutsugamushi* and *Anaplasma phagocytophilum* DNA in 14.51% and 5.57% of patients, and 10.96% of scrub typhus cases showed co-detection, emphasizing the need for reliable molecular discrimination among co-circulating agents [31]. From a genomic epidemiology perspective, patient-centered typing across Hainan has revealed at least 12 major *tsa56* genotypes, including lineages found in other parts of Southeast Asia with identical MLST sequence types across locations separated by 23–125 km, indicating within-island dissemination [20]. The regional distribution of related genotypes suggests that transmission connectivity may extend beyond strictly local rodent–chigger cycles. Because several of these genotypes are prevalent along the East Asian–Australasian flyway and multiple genetic-diversity hotspots overlap protected wetlands used by wintering waterbirds, migratory hosts that carry trombiculid mites warrant consideration as potential contributors to longer-range dispersal [20]. Coordinated sampling across humans, small mammals, mites, and bird-associated habitats coupled with genome-wide sequencing with help to address this hypothesis. In this setting, HMU_001 provides a closed genome for the TA763_A lineage from a recent clinical case, strengthening the reference framework for molecular surveillance and future studies integrating clinical metadata with genome-wide variation.

We further paired the closed genome of HMU_001 with a set of standardized in vitro measurements to provide biological context for genome-scale comparisons. In cell culture, HMU_001 showed the characteristic pleomorphic intracellular forms and perinuclear clustering consistent with the established intracellular lifecycle of *O. tsutsugamushi*. Using a qPCR-based assay, we quantified replication in endothelial cells and estimated a minimum doubling time of 9.36 ± 1.78 h. Previous reported qPCR-derived doubling times for diverse strains span approximately 9.50–19.85 h under fibroblast-based assays [46]. However, because kinetic estimates are highly dependent on host cell type and assay design, and HMU_001 was measured here in HUVECs, these published values are provided only as a general reference range and should not be interpreted as directly comparable to the present measurements. This caution is consistent with experimental evidence that cellular tropism and disease manifestations can vary across models [47,48], and that in vitro growth does not necessarily predict in vivo pathogenicity, which likely reflects a multifactorial interplay between bacterial determinants and host responses [21]. Nevertheless, pairing a contemporary clinical isolate with both phenotyping and a closed genome enables testable, multi-strain comparisons. Such studies can assess whether genome architecture, particularly repeat burden and mobile element content, is associated with replication dynamics in relevant cell types, dissemination phenotypes, or clinical severity.

Comparative analyses across the 17 complete genomes provide a framework for interpreting *O. tsutsugamushi* genome evolution. Despite marked genome size heterogeneity among *O. tsutsugamushi* strains, the nonrepetitive backbone remained constrained at approximately 1.00 Mb across strains, approached the size range typical of other compact genomes in *Rickettsiales* [27]. In contrast, differences in total genome size were explained primarily by expansion and contraction of repetitive DNA. A broadly comparable pattern has been reported in other *Rickettsiales*, including *Rickettsia buchneri,* and *Wolbachia pipientis* (family *Anaplasmataceae*). *R. buchneri* harbors a repeat fraction on the order of ~35% in a 1,777,907-bp genome, whereas *W. pipientis* strain wMel contains a much lower repeat fraction (~14.2%) in a streamlined 1,267,782-bp genome [49,50]. In this context, HMU_001 represents a lower-repeat endpoint within *O. tsutsugamushi* (46.08%) and, among the 17 complete genomes analyzed in this study, the most compact genome (1,895,724 bp). Its smaller size therefore tracks with a reduced repetitive fraction rather than with substantial loss of conserved functional gene content. Accordingly, HMU_001 shares broadly similar COG category composition with other strains, while most interstrain differences reflect copy-number variation within repeat-enriched categories instead of gain or loss of entire functional repertoires. This pattern aligns with pan-genome analyses suggesting that diversification in *O. tsutsugamushi* is driven in part by duplication and divergence of existing genes rather than extensive acquisition of novel pathways [51].

The same repeat landscape provides a mechanistic basis for pervasive genome rearrangement. In *O. tsutsugamushi* genomes, repeats are dominated by RAGEs, which encode an F-type T4SS, and by IS elements, both of which likely generate abundant homologous substrates for non-allelic recombination and structural change. RAGEs are conceptually related to F plasmid-like conjugation systems but exist as chromosomally integrated elements in *O. tsutsugamushi*. RAGE-like elements also occur in other *Rickettsiaceae*, including *Rickettsia bellii*, *Rickettsia massiliae*, *Rickettsia felis*, and *Rickettsia buchneri*. However, unlike the extensive amplification and degeneration seen in *O. tsutsugamushi*, these species typically carry only one or a few intact or near-intact elements [49,52]. Beyond integrases and Tra/Trb modules, RAGE regions commonly carry diverse cargo genes including multicopy ankyrin- and TPR-repeat proteins, several of which have been experimentally implicated in host-pathway manipulation [53,54,55]. In HMU_001, most RAGEs and many insertion sequences are fragmented, and even the most complete RAGE candidates show disrupted mobilization modules, indicating substantial decay. Nevertheless, intact copies of key mobilization genes persist in a dispersed configuration, and the high density of homologous repeats could continue to promote recombination-driven rearrangements even if only a minority of elements retains residual mobility [28]. Consistent with an expansion–degeneration cycle, domain-based inspection (SMART) and pseudogene prediction indicate that repetitive genes are disproportionately prone to pseudogenization compared with nonrepetitive genes, as previously noted for strain Ikeda [27], and more broadly across *Rickettsiales*, where pseudogenes can arise through progressive degradation of ancestral genes as well as rapid decay of genes gained by horizontal transfer or duplication [56]. Notably, pseudogenization rates are relatively similar among non-repetitive genes across strains, but are much higher and more variable among repetitive genes. Together, these observations suggest that strain-specific patterns of repeat proliferation and degeneration, rather than expansion or contraction of the conserved backbone, may represent an important feature of intraspecific genome evolution in *O. tsutsugamushi*.

Genome-wide phylogenies provide a useful framework for interpreting strain relatedness and broad patterns of genetic clustering in *O. tsutsugamushi*. In our orthogroup analysis, 95.5% of genes clustered into shared orthogroups, yet only 664 orthogroups were universally present and 643 were single-copy in all genomes, underscoring a relatively constrained core against a large and dynamic accessory complement. The maximum-likelihood phylogeny inferred from these 643 single-copy core genes resolved four clades and placed HMU_001 with Wuj/2014 from Zhejiang, TW-1 from Taiwan, and UT76 from Thailand, whereas the remaining clades grouped with strains from South Asia, Japan, Korea, and the India–Myanmar border region, indicating a geographic signal that is present but incomplete. However, geographic interpretation should remain cautious, because no formal population-structure analyses were performed, and phylogeographic inference is currently limited by sparse genome sampling and extensive recombination. The *tsa56* genotyping, the most widely used scheme for strain classification, was only partly congruent with the core-genome clades in our dataset. This discordance is biologically plausible for an immunodominant surface antigen subject to locus-specific recombination and selection, and it reinforces the limitations of single-locus classification in a highly recombinant species. When closed genomes are not available, an immediately testable interim approach is augmenting TSA56 with additional antigen loci; by which concatenated ScaA and TSA56 amino acid sequences might be an alternative option to better reflect the true situation of genome-scale phylogeny than TSA56 alone [25]. By adding a closed genome for the TA763_A lineage, HMU_001 strengthens the reference framework for interpreting *tsa56*-based calls and supports continued expansion of complete genomes across underrepresented regions and genotypes to improve molecular epidemiology and inference of strain movement.

Beyond variation in repeat burden and pseudogenization, genome structure is exceptionally labile in *O. tsutsugamushi*. Although other intracellular bacteria such as *Wolbachia pipientis* also harbored abundant mobile genetic elements and undergo genome rearrangement, the extent of erosion is substantially greater in *O. tsutsugamushi* [50]. In contrast, many *Rickettsia* species maintain compact, relatively stable genomes with conserved gene order, with notable exceptions such as *Rickettsia bellii* and *Rickettsia felis* [57,58]. Earlier complete-genome studies and subsequent long-read comparisons have emphasized a mosaic architecture in which core gene islands were interleaved with large repeat blocks, with disorder in the arrangement of the core gene islands across strains at the same time [22,28].

Our pairwise synteny analyses extend this framework quantitatively across the 17 closed genomes. HMU_001 shared the highest syntenic fractions with its closest relatives UT76, TW-1, and Wuj/2014, yet these alignments were broken into over a hundred syntenic blocks, indicating that numerous rearrangement breakpoints can accumulate even within a single clade. At the species scale, appreciable collinearity was restricted to near-identical pairs such as JJOtsu6–JJOtsu1 and Wuj/2014–TW-1, which retained nearly complete syntenic coverage but still differed by dozens of blocks. In contrast, the most distant comparisons, such as Boryong–TA686, retained only about one third syntenic sequence and were fragmented into more than a hundred of blocks. Moreover, the relationship between syntenic fraction and block count formed an almost continuous gradient across most genome pairs rather than discrete strata, supporting a model of cumulative, ongoing remodeling rather than rare episodic “scrambling” events. These patterns are consistent with repeated non-allelic homologous recombination between abundant repeat copies, many of which are derived from RAGE and IS elements, plausibly contributing to inversions, translocations, and segment loss while leaving the conserved backbone comparatively intact. This degree of structural turnover complicates comparative inference because genomic context and conserved-island boundaries are frequently not transferable between strains and are best assessed using closed genomes. At the same time, highly syntenic strain pairs provide informative snapshots of early divergence, offering an opportunity to quantify how rapidly rearrangements accumulate and to test whether structural trajectories correlate with local transmission dynamics or strain-level phenotypes.

Finally, our study highlights a practical way to generate closed *O. tsutsugamushi* genomes while reducing hands-on manipulation. The extreme repeat content of *O. tsutsugamushi* complicates closure with short-read data, and long-read approaches depend on careful DNA preparation and sufficiently long molecules to span repeat regions. Earlier workflows often relied on extensive enrichment or purification from host cells, including density-gradient methods, or enzymatic treatment to eliminate residual host DNA to obtain high-quality bacterial genomic DNA material. In contrast, we sequenced HMU_001 as a nonclonal isolate without plaque purification or density-gradient enrichment, combining deep long-read sequencing with stringent host read removal and short-read polishing to obtain a closed genome, and we supported within-isolate homogeneity using genome-wide allele-frequency profiles and stability of *tsa* loci across passages. This reduced handling is operationally relevant because *O. tsutsugamushi* propagation is technically demanding and typically requires biosafety level 3 facilities. In parallel, probe-based target enrichment strategies from human and chigger samples have been developed to address low pathogen DNA and the technical, safety, and cost constraints of cell culture, enabling genome-scale phylogenetic analyses when culture or genome closure is not feasible [59]. Together, reduced-purification culture-to-genome workflows and culture-independent enrichment should help build denser, geographically representative genome panels for surveillance and for testing how repeat-mediated genome remodeling relates to transmission and disease.

Our study has limitations. First, our inferences about genome compaction and repeat dynamics are based on a single contemporary isolate, so the full range of repeat burden and RAGE configurations circulating in Hainan remains to be defined. Second, variation in repeat burden, RAGE integrity, and putative effector repertoires was inferred from sequence and domain annotations, and the biological consequences of these differences require experimental validation. Third, even with standardized re-annotation, repeat-dense regions remain difficult to annotate and compare consistently across studies, which can influence gene and pseudogene estimates. Despite these caveats, HMU_001 provided a high-quality closed genome from a recent clinical isolate in a high-incidence setting, and strengthened the empirical foundation for linking repeat proliferation, genome rearrangement, and strain typing in *O. tsutsugamushi*.

## Figures and Tables

**Table 1:** Summary of *Orientia tsutsugamushi* (*O. tsutsugamushi*) strains with complete genome.

No.	Strain Name	Geographic Location	Host Source	Collection Date	Release Date	Size (bp)	Gene *	CDS *	tRNA *	rRNA *	Pseudogene *	*tsa*56 Genotype #	Sequence Type (ST)	Reference
1	HMU_001	Hainan	*Homo sapiens*	2023	/	1,895,724	2075	2038	34	3	765	TA763_A	NA ^†^	this study
2	JJOtsu7	Vellore	*Homo sapiens*	2024	22 August 2024	2,183,885	2462	2425	34	3	987	Karp_A	133	unpublished
3	JJOtsu8	Vellore	*Homo sapiens*	2023	22 August 2024	2,344,138	2592	2554	35	3	914	TA763_B	NA ^†^	unpublished
4	JJOtsu5	Vellore	*Homo sapiens*	2023	22 August 2024	2,446,845	2746	2709	34	3	1019	Karp_A	135	unpublished
5	JJOtsu6	Vellore	*Homo sapiens*	2023	22 August 2024	2,284,757	2523	2487	33	3	955	Kato_A	132	unpublished
6	JJOtsu1	Vellore	*Homo sapiens*	2022	22 August 2024	2,179,789	2447	2410	34	3	963	Kato_A	132	unpublished
7	Wuj/2014	Zhejiang	*Homo sapiens*	2014	25 September 2019	1,972,387	2148	2111	34	3	831	Karp_A	NA ^†^	unpublished
8	TW-1	Taiwan	*Homo sapiens*	2007	1 May 2024	2,008,429	2150	2113	34	3	806	Karp_A	NA ^†^	[25] Minahan NT et al. 2024.
9	TW-22	Taiwan	*Homo sapiens*	2007	1 May 2024	2,044,475	2315	2278	34	3	869	Kato_A	NA ^†^	[25] Minahan NT et al. 2024.
10	UT176	Udon Thani	*Homo sapiens*	2004	12 May 2018	1,932,116	2124	2086	35	3	838	Karp_B	10	[22] Batty EM et al. 2018.
11	UT76	Udon Thani	*Homo sapiens*	2003	12 May 2018	2,078,193	2284	2247	34	3	863	Karp_A	1	[22] Batty EM et al. 2018.
12	Boryong	South Korea	*Homo sapiens*	1980s	15 May 2007	2,127,051	2480	2443	34	3	1088	Boryong/Karp	48	[26] Cho NH et al. 2007.
13	Ikeda	Japan	*Homo sapiens*	1979	30 May 2008	2,008,987	2222	2185	34	3	882	JG_A/Gilliam	49	[27] Nakayama K et al. 2008.
14	TA686	Thailand	*Tupaia glis*	1963	12 May 2018	2,254,553	2583	2546	34	3	1122	Shimokoshi	NA ^†^	[22] Batty EM et al. 2018.
15	Kato	Niigata	*Homo sapiens*	1955	12 May 2018	2,319,449	2444	2406	35	3	863	Kato_B	20	[22] Batty EM et al. 2018.
16	Karp	New Guinea	*Homo sapiens*	1943	12 May 2018	2,469,803	2615	2578	34	3	943	Karp_C	45	[22] Batty EM et al. 2018.
17	Gilliam	Indian-Burmese border	*Homo sapiens*	1943	12 May 2018	2,465,012	2746	2709	34	3	1030	Gilliam	46	[22] Batty EM et al. 2018.

* To ensure consistency with this study, genomes retrieved from Genebank were re-annotated for genes, CDSs, tRNAs, rRNAs and pseudogenes following the same procedure described in Materials and Methods. # *tsa56* genotypes were classified based on phylogenetic tree derived from partial sequences of *tsa56* genes. ^†^ NA indicates that the seven-locus MLST allele profile had not been assigned an ST in the PubMLST *O. tsutsugamushi* database at the time of analysis.

## Data Availability

The complete genome sequence of *O. tsutsugamushi* strain HMU_001 has been deposited in NCBI datasets under accession CP191261 (BioProject: PRJNA1258383, BioSample: SAMN48320726). Raw sequencing reads have been deposited in the NCBI Sequence Read Archive under accession SRR33649153 (PacBio) and SRR33649152 (MGI).

## Supplementary Materials

The following supporting information can be downloaded at: https://www.mdpi.com/article/10.3390/pathogens15030318/s1, Table S1: Results of laboratory examinations; Table S2: Overall annotations of genes in HMU_001 genome; Table S3: Category of pseudogenes in HMU_001 genome; Table S4: Gene numbers and repeat ratios in each COG category; Table S5: Locations of repeat regions in HMU_001 genome; Table S6: Repeat and non-repeat size; Table S7: Pseudogenization in repeat and non-repeat genes; Table S8: Annotations of RAGEs in HMU_001 genome; Table S9: Annotations of insertion sequences in HMU_001 genome; Table S10: Sequence type (ST) of strains analysed in this study; Table S11: Counts and ratios of syntenic blocks in each pair of genomes; Figure S1: Sequencing depth distribution histogram and genome-wide profile; Figure S2: Gene organization of RAGE loci with canonical features in HMU_001; Figure S3: Phylogenetic relationships inferred from partial *tsa56* sequences.

## References

[1] Bhandari M., Singh R.K., Laishevtcev A., Mohapatra T.M., Nigam M., Mori E., Vasconcelos de Lacerda B.C.G., Coutinho H.D.M., Mishra A.P. (2022). Revisiting scrub typhus: A neglected tropical disease. Comp. Immunol. Microbiol. Infect. Dis..

[2] Xu G., Walker D.H., Jupiter D., Melby P.C., Arcari C.M. (2017). A review of the global epidemiology of scrub typhus. PLoS Negl. Trop. Dis..

[3] Walker D.H. (2016). Scrub Typhus-Scientific Neglect, Ever-Widening Impact. N. Engl. J. Med..

[4] Jiang J., Richards A.L. (2018). Scrub Typhus: No Longer Restricted to the Tsutsugamushi Triangle. Trop. Med. Infect. Dis..

[5] Alkathiry H.A., Alghamdi S.Q., Morgan H.E.J., Noll M.E., Khoo J.J., Alagaili A.N., Makepeace B.L. (2023). Molecular Detection of Candidatus Orientia chuto in Wildlife, Saudi Arabia. Emerg. Infect. Dis..

[6] Abarca K., Martínez-Valdebenito C., Angulo J., Jiang J., Farris C.M., Richards A.L., Acosta-Jamett G., Weitzel T. (2020). Molecular Description of a Novel Orientia Species Causing Scrub Typhus in Chile. Emerg. Infect. Dis..

[7] Dasgupta S., Asish P.R., Rachel G., Bagepally B.S., Chethrapilly Purushothaman G.K. (2024). Global seroprevalence of scrub typhus: A systematic review and meta-analysis. Sci. Rep..

[8] Gaba S., Gupta M., Singla N., Singh R. (2019). Clinical outcome and predictors of severity in scrub typhus patients at a tertiary care hospital in Chandigarh, India. J. Vector Borne Dis..

[9] Kumar V., Kumar V., Yadav A.K., Iyengar S., Bhalla A., Sharma N., Aggarwal R., Jain S., Jha V. (2014). Scrub typhus is an under-recognized cause of acute febrile illness with acute kidney injury in India. PLoS Negl. Trop. Dis..

[10] Saraswati K., Maguire B.J., McLean A.R.D., Singh-Phulgenda S., Ngu R.C., Newton P.N., Day N.P.J., Guérin P.J. (2021). Systematic review of the scrub typhus treatment landscape: Assessing the feasibility of an individual participant-level data (IPD) platform. PLoS Negl. Trop. Dis..

[11] Blacksell S.D., Bryant N.J., Paris D.H., Doust J.A., Sakoda Y., Day N.P. (2007). Scrub typhus serologic testing with the indirect immunofluorescence method as a diagnostic gold standard: A lack of consensus leads to a lot of confusion. Clin. Infect. Dis..

[12] Kannan K., John R., Kundu D., Dayanand D., Abhilash K.P.P., Mathuram A.J., Zachariah A., Sathyendra S., Hansdak S.G., Abraham O.C. (2020). Performance of molecular and serologic tests for the diagnosis of scrub typhus. PLoS Negl. Trop. Dis..

[13] Taylor A.J., Paris D.H., Newton P.N. (2015). A Systematic Review of Mortality from Untreated Scrub Typhus (*Orientia tsutsugamushi*). PLoS Negl. Trop. Dis..

[14] Wang Q., Ma T., Ding F., Lim A., Takaya S., Saraswati K., Sartorius B., Day N.P.J., Maude R.J. (2024). Global and regional seroprevalence, incidence, mortality of, and risk factors for scrub typhus: A systematic review and meta-analysis. Int. J. Infect. Dis..

[15] Kelly D.J., Fuerst P.A., Richards A.L. (2019). Origins, Importance and Genetic Stability of the Prototype Strains Gilliam, Karp and Kato of *Orientia tsutsugamushi*. Trop. Med. Infect. Dis..

[16] Kang J.S., Chang W.H. (1999). Antigenic relationship among the eight prototype and new serotype strains of *Orientia tsutsugamushi* revealed by monoclonal antibodies. Microbiol. Immunol..

[17] Furuya Y., Yoshida Y., Katayama T., Yamamoto S., Kawamura A. (1993). Serotype-specific amplification of *Rickettsia tsutsugamushi* DNA by nested polymerase chain reaction. J. Clin. Microbiol..

[18] Sihag K.K., Arif W., Srirama S., Chandrasekaran A.K., Raveendran V., Chandrakumar A.B., Kasirajan A., Thavaraj S.A.P., Srinivasan L., Choolayil A.C. (2025). A longitudinal molecular surveillance of genetic heterogeneity of *Orientia tsutsugamushi* in humans, reservoir animals, and vectors in Puducherry, India. Front. Microbiol..

[19] Wang G., Fu R., Zhang L., Xue L., Al-Mahdi A.Y., Xie X., Qin A., Tang C., Du J., Huang Y. (2023). Genomic bacterial load associated with bacterial genotypes and clinical characteristics in patients with scrub typhus in Hainan Island, Southern China. PLoS Negl. Trop. Dis..

[20] Tang C., Huang Y., Wang G., Xue L., Hu X., Peng R., Du J., Yang J., Niu Y., Deng W. (2025). Patient-centric analysis of *Orientia tsutsugamushi* spatial diversity patterns across Hainan Island, China. PLoS Negl. Trop. Dis..

[21] Chaichana P., Satapoomin N., Kullapanich C., Chuenklin S., Mohammad A., Inthawong M., Ball E.E., Burke T.P., Sunyakumthorn P., Salje J. (2025). Comparative virulence analysis of seven diverse strains of *Orientia tsutsugamushi* reveals a multifaceted and complex interplay of virulence factors responsible for disease. PLoS Pathog..

[22] Batty E.M., Chaemchuen S., Blacksell S., Richards A.L., Paris D., Bowden R., Chan C., Lachumanan R., Day N., Donnelly P. (2018). Long-read whole genome sequencing and comparative analysis of six strains of the human pathogen *Orientia tsutsugamushi*. PLoS Negl. Trop. Dis..

[23] Sonthayanon P., Peacock S.J., Chierakul W., Wuthiekanun V., Blacksell S.D., Holden M.T., Bentley S.D., Feil E.J., Day N.P. (2010). High rates of homologous recombination in the mite endosymbiont and opportunistic human pathogen *Orientia tsutsugamushi*. PLoS Negl. Trop. Dis..

[24] Phetsouvanh R., Sonthayanon P., Pukrittayakamee S., Paris D.H., Newton P.N., Feil E.J., Day N.P. (2015). The Diversity and Geographical Structure of *Orientia tsutsugamushi* Strains from Scrub Typhus Patients in Laos. PLoS Negl. Trop. Dis..

[25] Minahan N.T., Yen T.Y., Guo Y.L., Shu P.Y., Tsai K.H. (2024). Concatenated ScaA and TSA56 Surface Antigen Sequences Reflect Genome-Scale Phylogeny of *Orientia tsutsugamushi*: An Analysis Including Two Genomes from Taiwan. Pathogens.

[26] Cho N.H., Kim H.R., Lee J.H., Kim S.Y., Kim J., Cha S., Kim S.Y., Darby A.C., Fuxelius H.H., Yin J. (2007). The *Orientia tsutsugamushi* genome reveals massive proliferation of conjugative type IV secretion system and host-cell interaction genes. Proc. Natl. Acad. Sci. USA.

[27] Nakayama K., Yamashita A., Kurokawa K., Morimoto T., Ogawa M., Fukuhara M., Urakami H., Ohnishi M., Uchiyama I., Ogura Y. (2008). The Whole-genome sequencing of the obligate intracellular bacterium *Orientia tsutsugamushi* revealed massive gene amplification during reductive genome evolution. DNA Res..

[28] Giengkam S., Kullapanich C., Wongsantichon J., Adcox H.E., Gillespie J.J., Salje J. (2023). *Orientia tsutsugamushi*: Comprehensive analysis of the mobilome of a highly fragmented and repetitive genome reveals the capacity for ongoing lateral gene transfer in an obligate intracellular bacterium. mSphere.

[29] Nakano K., Shiroma A., Shimoji M., Tamotsu H., Ashimine N., Ohki S., Shinzato M., Minami M., Nakanishi T., Teruya K. (2017). Advantages of genome sequencing by long-read sequencer using SMRT technology in medical area. Hum. Cell.

[30] Liu P.Y., Jia P.B., Chen L., Jin Y.M., Feng F.L., Pan B.Y., Shen Y.M., Lin X.Z., He Y.N. (2024). Epidemiological characteristics and trend prediction of scrub typhus in Hainan Province from 2008 to 2023. China Trop. Med..

[31] Xie X., Zhang Y., Teng Z., Duan B., Hai Y., Wang M., Shao Z., Liang W., Kan B., Yin F. (2024). The Prevalence of Rickettsial and Rickettsial-Like Diseases in Patients with Undifferentiated Febrile Illness-Hainan Province, China, 2018–2021. China CDC Wkly..

[32] Kim D.M., Park G., Kim H.S., Lee J.Y., Neupane G.P., Graves S., Stenos J. (2011). Comparison of conventional, nested, and real-time quantitative PCR for diagnosis of scrub typhus. J. Clin. Microbiol..

[33] Giengkam S., Blakes A., Utsahajit P., Chaemchuen S., Atwal S., Blacksell S.D., Paris D.H., Day N.P., Salje J. (2015). Improved Quantification, Propagation, Purification and Storage of the Obligate Intracellular Human Pathogen *Orientia tsutsugamushi*. PLoS Negl. Trop. Dis..

[34] Cheng H., Concepcion G.T., Feng X., Zhang H., Li H. (2021). Haplotype-resolved de novo assembly using phased assembly graphs with hifiasm. Nat. Methods.

[35] Hunt M., Silva N.D., Otto T.D., Parkhill J., Keane J.A., Harris S.R. (2015). Circlator: Automated circularization of genome assemblies using long sequencing reads. Genome Biol..

[36] Walker B.J., Abeel T., Shea T., Priest M., Abouelliel A., Sakthikumar S., Cuomo C.A., Zeng Q., Wortman J., Young S.K. (2014). Pilon: An integrated tool for comprehensive microbial variant detection and genome assembly improvement. PLoS ONE.

[37] Seemann T. (2014). Prokka: Rapid prokaryotic genome annotation. Bioinformatics.

[38] Nawrocki E.P., Eddy S.R. (2013). Infernal 1.1: 100-fold faster RNA homology searches. Bioinformatics.

[39] Chan P.P., Lowe T.M. (2019). tRNAscan-SE: Searching for tRNA Genes in Genomic Sequences. Gene Prediction.

[40] Syberg-Olsen M.J., Garber A.I., Keeling P.J., McCutcheon J.P., Husnik F. (2022). Pseudofinder: Detection of Pseudogenes in Prokaryotic Genomes. Mol. Biol. Evol..

[41] Cantalapiedra C.P., Hernández-Plaza A., Letunic I., Bork P., Huerta-Cepas J. (2021). eggNOG-mapper v2: Functional Annotation, Orthology Assignments, and Domain Prediction at the Metagenomic Scale. Mol. Biol. Evol..

[42] Emms D.M., Kelly S. (2019). OrthoFinder: Phylogenetic orthology inference for comparative genomics. Genome Biol..

[43] Stamatakis A. (2014). RAxML version 8: A tool for phylogenetic analysis and post-analysis of large phylogenies. Bioinformatics.

[44] Jolley K.A., Bray J.E., Maiden M.C.J. (2018). Open-access bacterial population genomics: BIGSdb software, the PubMLST.org website and their applications. Wellcome Open Res..

[45] Marçais G., Delcher A.L., Phillippy A.M., Coston R., Salzberg S.L., Zimin A. (2018). MUMmer4: A fast and versatile genome alignment system. PLoS Comput. Biol..

[46] Phuklia W., Panyanivong P., Sengdetka D., Sonthayanon P., Newton P.N., Paris D.H., Day N.P.J., Dittrich S. (2019). Novel high-throughput screening method using quantitative PCR to determine the antimicrobial susceptibility of *Orientia tsutsugamushi* clinical isolates. J. Antimicrob. Chemother..

[47] Inthawong M., Sunyakumthorn P., Wongwairot S., Anantatat T., Dunachie S.J., Im-Erbsin R., Jones J.W., Mason C.J., Lugo L.A., Blacksell S.D. (2022). A time-course comparative clinical and immune response evaluation study between the human pathogenic *Orientia tsutsugamushi* strains: Karp and Gilliam in a rhesus macaque (*Macaca mulatta*) model. PLoS Negl. Trop. Dis..

[48] Thiriot J.D., Liang Y., Gonzales C., Sun J., Yu X., Soong L. (2023). Differential cellular immune responses against *Orientia tsutsugamushi* Karp and Gilliam strains following acute infection in mice. PLoS Negl. Trop. Dis..

[49] Hagen R., Verhoeve V.I., Gillespie J.J., Driscoll T.P. (2018). Conjugative Transposons and Their Cargo Genes Vary across Natural Populations of *Rickettsia buchneri* Infecting the Tick *Ixodes scapularis*. Genome Biol. Evol..

[50] Wu M., Sun L.V., Vamathevan J., Riegler M., Deboy R., Brownlie J.C., McGraw E.A., Martin W., Esser C., Ahmadinejad N. (2004). Phylogenomics of the reproductive parasite *Wolbachia pipientis* wMel: A streamlined genome overrun by mobile genetic elements. PLoS Biol..

[51] Fleshman A., Mullins K., Sahl J., Hepp C., Nieto N., Wiggins K., Hornstra H., Kelly D., Chan T.C., Phetsouvanh R. (2018). Comparative pan-genomic analyses of *Orientia tsutsugamushi* reveal an exceptional model of bacterial evolution driving genomic diversity. Microb. Genom..

[52] Gillespie J.J., Joardar V., Williams K.P., Driscoll T., Hostetler J.B., Nordberg E., Shukla M., Walenz B., Hill C.A., Nene V.M. (2012). A *Rickettsia* genome overrun by mobile genetic elements provides insight into the acquisition of genes characteristic of an obligate intracellular lifestyle. J. Bacteriol..

[53] Adcox H.E., Hunt J.R., Allen P.E., Siff T.E., Rodino K.G., Ottens A.K., Carlyon J.A. (2024). *Orientia tsutsugamushi* Ank5 promotes NLRC5 cytoplasmic retention and degradation to inhibit MHC class I expression. Nat. Commun..

[54] Evans S.M., Rodino K.G., Adcox H.E., Carlyon J.A. (2018). *Orientia tsutsugamushi* uses two Ank effectors to modulate NF-κB p65 nuclear transport and inhibit NF-κB transcriptional activation. PLoS Pathog..

[55] Bang S., Min C.K., Ha N.Y., Choi M.S., Kim I.S., Kim Y.S., Cho N.H. (2016). Inhibition of eukaryotic translation by tetratricopeptide-repeat proteins of *Orientia tsutsugamushi*. J. Microbiol..

[56] Fuxelius H.H., Darby A.C., Cho N.H., Andersson S.G. (2008). Visualization of pseudogenes in intracellular bacteria reveals the different tracks to gene destruction. Genome Biol..

[57] Blanc G., Ogata H., Robert C., Audic S., Suhre K., Vestris G., Claverie J.M., Raoult D. (2007). Reductive genome evolution from the mother of *Rickettsia*. PLoS Genet..

[58] He M., Zhang L., Hu H., Liu X., Zhang C., Xin Y., Liu B., Chen Z., Xu K., Liu Y. (2023). Complete genome sequencing and comparative genomic analyses of a new spotted-fever *Rickettsia heilongjiangensis* strain B8. Emerg. Microbes Infect..

[59] Elliott I., Thangnimitchok N., de Cesare M., Linsuwanon P., Paris D.H., Day N.P.J., Newton P.N., Bowden R., Batty E.M. (2021). Targeted capture and sequencing of *Orientia tsutsugamushi* genomes from chiggers and humans. Infect. Genet. Evol..

