# Supplementary material for: A Compact Closed Genome of Orientia tsutsugamushi from Hainan Island, China Provides a TA763_A Reference and Reveals Repeat-Driven Remodeling
Source: Pathogens. 2026 Mar 16;15(3):318. doi: 10.3390/pathogens15030318 (PMC13029110; doi:10.3390/pathogens15030318)
Supplement: Supplementary file 1 [file pathogens-15-00318-s001.zip › 20260311_Figure S1-S3 final.pdf]

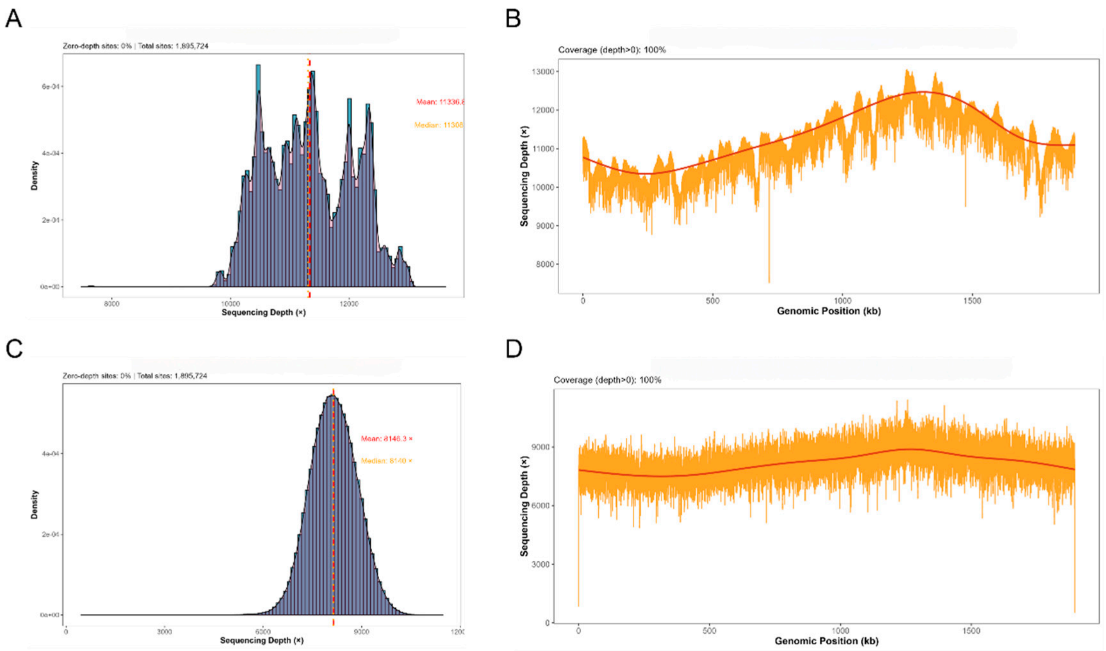

Figure S1. Sequencing depth distribution histogram and genome-wide profile.

Sequencing depth distribution histogram presenting density distribution of HiFi reads (A) and short paired end reads (C). Genome-wide sequencing depth profile presenting depth track along chromosomal coordinates for Hifi reads (B) and short paired-end reads (D).

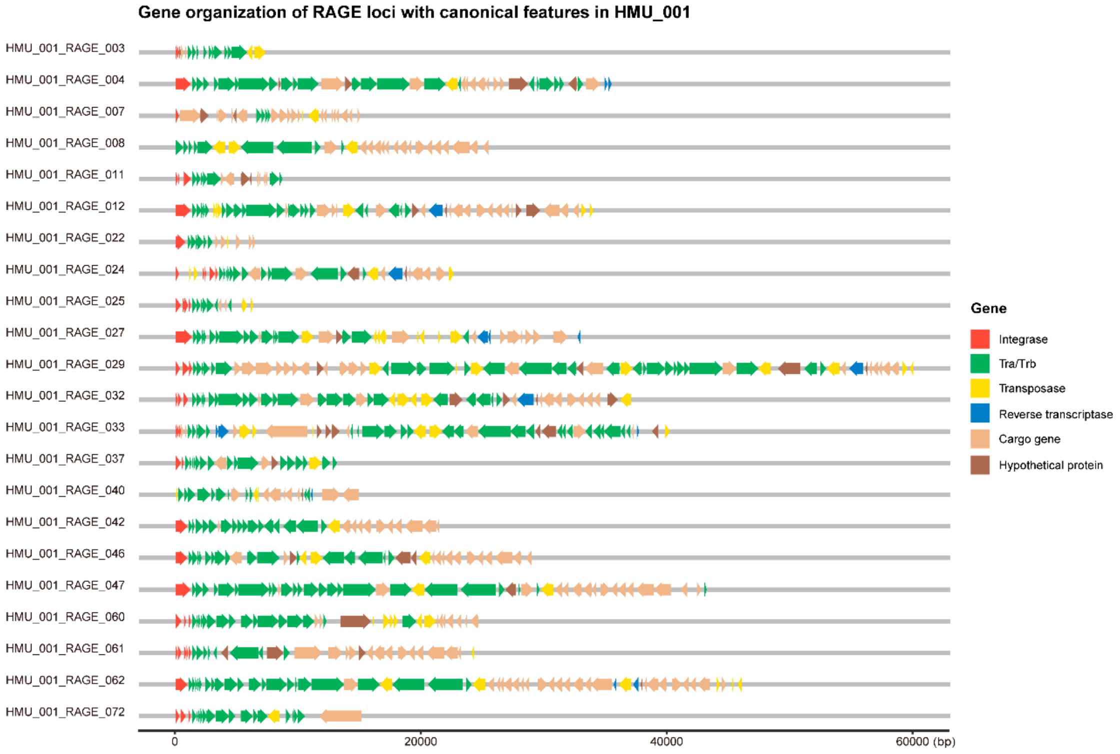

Figure S2. Gene organization of RAGE loci with canonical features in HMU_001.

Twenty-two RAGE loci that retain recognizable canonical components, including an integrase, *tra*/*trb* genes, and associated mobile-element and cargo genes. Arrows represent genes and are colored by module as in Figure 3D.

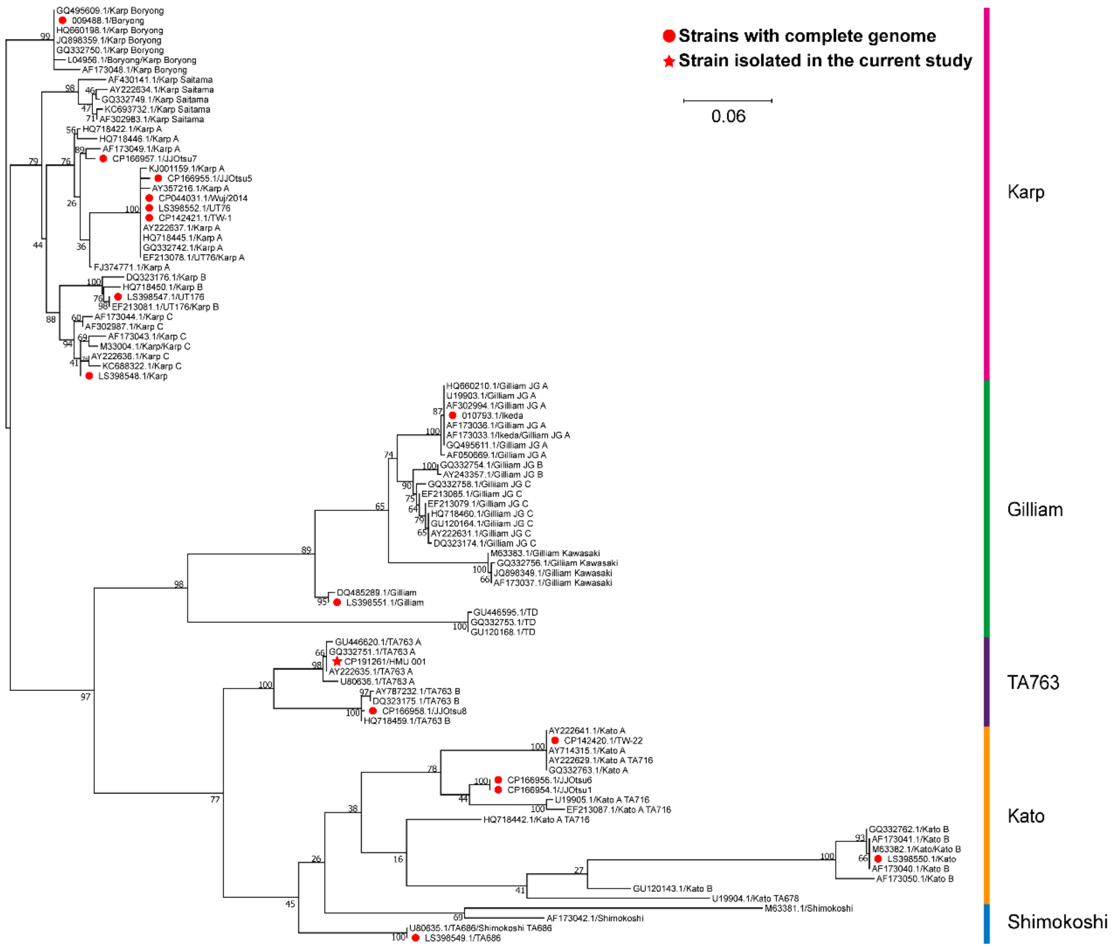

Figure S3. Phylogenetic relationships inferred from partial *tsa56* sequences.

Maximum-likelihood tree based on a partial *tsa56* alignment. Strains with complete genomes are marked with red circles, and HMU_001 is indicated by a red star. Colored bars indicate major *tsa56* genotype groups.
